# Supplementary material for: The causal relationship between the human gut microbiota and pyogenic arthritis: a Mendelian randomization study
Source: Front Cell Infect Microbiol. 2024 Nov 26;14:1452480. doi: 10.3389/fcimb.2024.1452480 (PMC11629706; doi:10.3389/fcimb.2024.1452480)
Supplement: Supplementary file 1 [file DataSheet1.zip › mendelian test/ebi-a-GCST90027613.csv_leave_one_out.pdf]

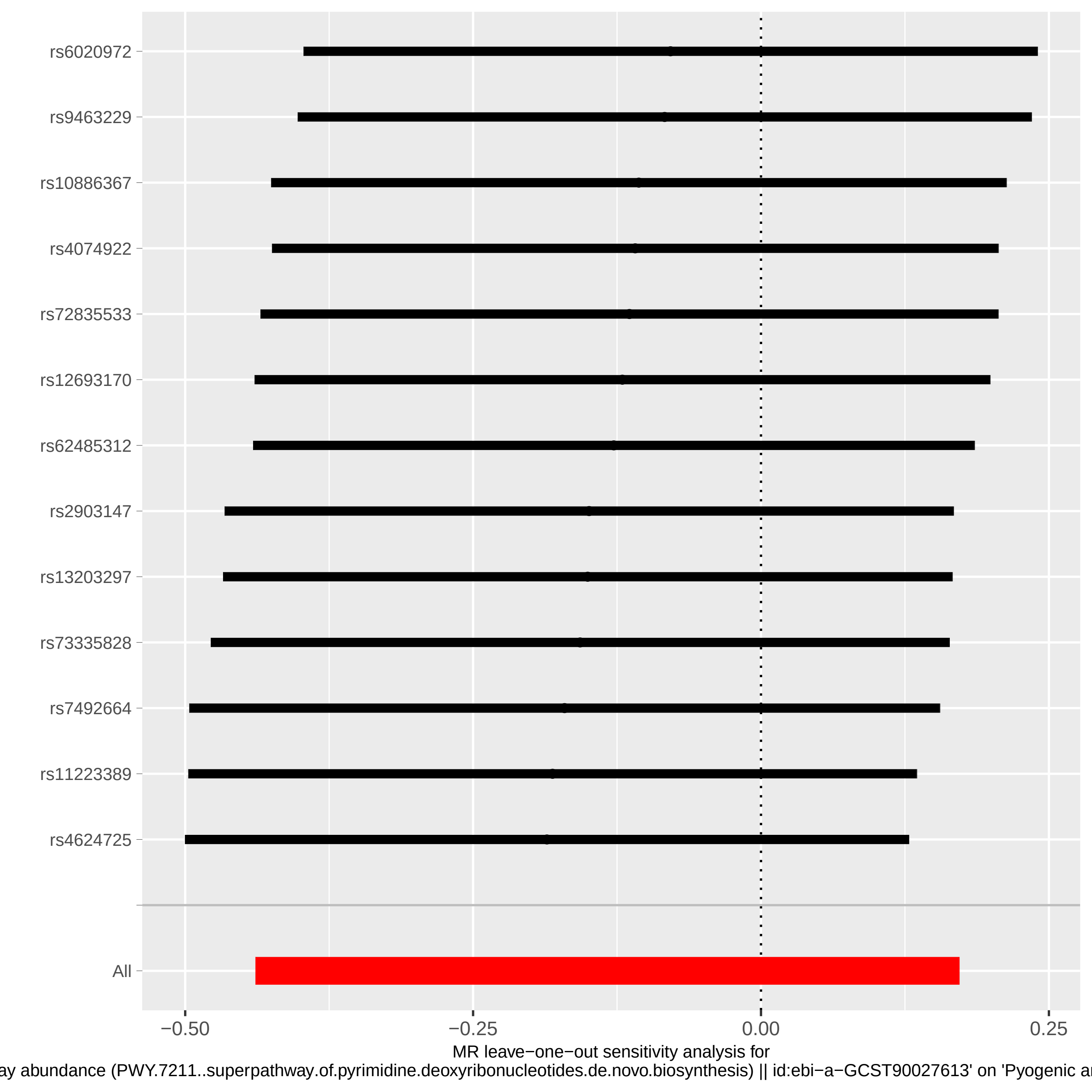
rs6020972
rs9463229
rs10886367
rs4074922
rs72835533
rs12693170
rs62485312
rs2903147
rs13203297
rs73335828
rs7492664
rs11223389
rs4624725
All
−0.50
−0.25
0.00
0.25
MR leave−one−out sensitivity analysis for
ay abundance (PWY.7211..superpathway.of.pyrimidine.deoxyribonucleotides.de.novo.biosynthesis) || id:ebi−a−GCST90027613' on 'Pyogenic a
